# A Newly Developed Variable Stiffness Duodenoscope for Diagnostic and Therapeutic Endoscopic Retrograde Cholangiopancreatography

**DOI:** 10.1155/2010/153951

**Published:** 2010-12-08

**Authors:** Takao Itoi, Atsushi Sofuni, Fumihide Itokawa, Toshio Kurihara, Takayoshi Tsuchiya, Kentaro Ishii, Shujiro Tsuji, Nobuhito Ikeuchi, Junko Umeda, Fuminori Moriyasu, Kazuhiko Kasuya, Akihiko Tsuchida

**Affiliations:** Department of Gastroenterology and Hepatology, Tokyo Medical University, 6-7-1 Nishishinjuku, Shinjuku-ku, Tokyo 160-0023, Japan

## Abstract

The aim of this study is to evaluate a prototype variable stiffness duodenoscope (VSD) for diagnostic and therapeutic ERCP in comparison with standard duodenoscopes. We performed retrospective analysis on the success rate of intubation of the second duodenum, overall procedural success rate, and comparative frequency of the necessity to change duodenoscopes from standard JF-260V and TJF 260V or to change stiffness using the VSD. A total of 213 nonconsecutive procedures in 196 patients with pancreaticobiliary diseases. There was no statistically significant difference in endoscope intubation rate or technical success rate between the different duodenoscopes. In one patient with severe duodenal stenosis, the VSD using the moderately stiff mode allowed the major papilla to be reached when the TJF-260V endoscope could not. There were no serious procedure-related adverse events. In conclusion, while the VSD performed well, the present models do not appear to offer obvious advantages over the standard duodenoscopes for routine diagnostic and therapeutic ERCP. Prospective studies may be warranted to identify those patients who would benefit from this new technology.

## 1. Introduction

The side-viewing duodenoscope was developed for endoscopic retrograde cholangiopancreatography (ERCP) more than 40 years ago [1, 2]. Since then, various duodenoscopes have been developed, such as a large-channel duodenoscope [3], an electronic video duodenoscope [4], a double-channel duodenoscope [5], and a duodenoscope with a modified v-shaped elevator (“V-scope”) that facilitates use of a short guidewire [6]. Recently, A prototype variable stiffness duodenoscope has been developed for ERCP. The concept is similar to what has been developed for colonoscopy. Variable stiffness colonoscopes have been found to be useful by reducing colonoscopy procedure time and procedure-related discomfort, with an increase in success rate of cecal intubation compared to conventional colonoscopes [7–10]. In the present study, we retrospectively evaluated a prototype variable stiffness duodenoscope for diagnostic and therapeutic ERCP and compared it with standard duodenoscopes.

## 2. Patients and Methods

A total of 213 nonconsecutive ERCPs (196 patients; 116 men, 80 women; with 17 repeat examinations) were performed during a 4-month period (September 2008 to January 2009) at the Tokyo Medical University Hospital. These cases were retrospectively reviewed. Patients with known esophageal stricture, prior Billroth-II gastrectomy, and Roux-en-Y anastomosis were excluded. All procedures were performed by five endoscopists (T.I., A.S., F.I., K.I., and S.T.), each of who had performed more than 500 ERCPs.

**Figure 1:**
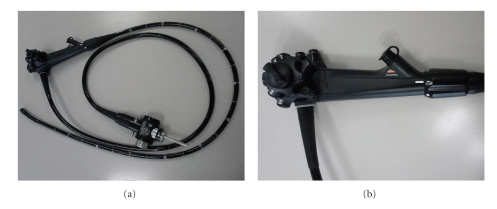
(a) Prototype variable stiffness duodenoscope. (b) The stiffness of the endoscope can be varied using the stiffness control ring at the base of the control section.

**Figure 2:**
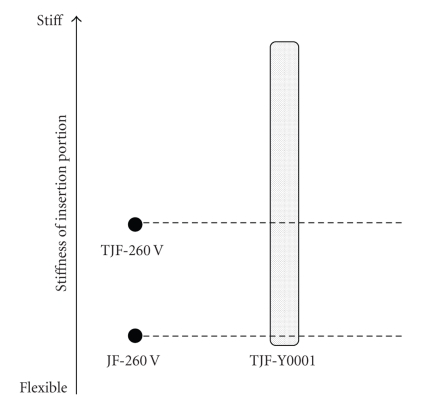
This figure was provided by the manufacture and demonstrates the relative flexibility (there are no units) compared to standard duodenoscopes (JF-260V and TJF-260V).

The prototype variable stiffness video duodenoscope TJF-Y0001 (Figure 1(a), Olympus Medical Systems, Tokyo, Japan) was developed based on the TJF-260V duodenoscope (Olympus). The prototype duodenoscope has the same basic specifications as the TJF-260 V but with a slightly larger outer diameter (11.6 mm versus 11.3 mm) (Table 1). In addition, the insertion tube stiffness can be adjusted from a point 15 cm from the distal end of the insertion tube to the proximal end of the insertion tube. The degree of stiffness of the prototype endoscope is adjusted using a rotary stiffness-control ring located at the lower portion of the control section (Figure 1(b)). Mode 0 has the same stiffness as the JF-260V endoscope (Olympus). Mode 1 has the same stiffness as the TJF-260V endoscope (Olympus). The stiffness of mode 2 is between those of mode 1 and 3. Mode 3 is the stiffest (Figure 2). Since we routinely use either the JF-260V or TJF-260V, or both, we elected to alternatively use the standard duodenoscopes and the prototype TJF-Y0001. When the TJF-Y0001 was used, the examination was begun using mode 0 stiffness, changing the degree of stiffness when difficulties in intubation and cannulation were encountered and during therapeutic maneuvers. If difficulties were encountered using the standard duodenoscopes the endoscope was changed to the TJF-Y0001 while using modes 1 or 2.

In all cases the following were recorded: (a) success rate of intubation of the second duodenum, (b) total success rate of procedure as defined by accomplishment of target destinations, and (c) necessity of changing endoscopes or stiffness of the prototype duodenoscope. The institutional review board of our institution approved this study. Written informed consent was obtained from all patients.

## 3. Statistical Analysis

Statistical analysis was performed by the chi-square test or Fisher's exact test for noncontinuous variables and the Student's *t*-test for continuous variables. A *P* value less than  .05 was regarded as being statistically significant. Statistical analyses were performed with StatMate III (ATMS Co Ltd, Tokyo, Japan).

## 4. Results

Findings in the 213 patients were biliary stones (95), benign pancreatobiliary strictures (21), chronic pancreatitis (31), pancreatic and biliary tumors (79), autoimmune pancreatitis (4), pancreatic pseudocyst (3), abnormal pancreaticobiliary junction (2), and other (7) cases. More than one of the above findings was present in 29 patients.

The number of ERCP cases using the TJF-Y0001, JF-260V, and TJF-260V were 86, 60, and 67, respectively. The characteristics of the patients undergoing examination or treatment using each type of duodenoscope are shown in Table 2. The numbers of cases of duodenal stenosis, in which the TJF-Y0001, JF-260V, and TJF-260V were used, were 4, 2, and 3, respectively. Procedures performed with each type of duodenoscope are shown in Table 3. The total number of therapeutic procedures performed using the TJF-Y0001, JF-260V, and TJF-260V were 170, 147, and 141, respectively. 

 The success rates of intubation of the second duodenum were 100%, 100%, and 98.5% for the TJF-Y0001, JF-260V, and TJF-260V endoscopes, respectively. There was no statistically significant difference. In one case of severe duodenal stenosis, the papilla could not be reached using the TJF-260V endoscope because it looped within the fundus of the stomach. In this case, the duodenoscope was changed to the TJF-Y0001, using mode 2, to successfully reach the papilla beyond the duodenal stenosis. 

With the exception of a single failed insertion using the TJF-260V endoscope, target destinations were achieved with all types of duodenoscopes. The number of cases in which precut sphincterotomy was performed using the TJF-Y0001, JF-260V, or TJF-260V endoscope was 2 (3.8%), 1 (2.9%), and 1 (2.4%), respectively. There was no adverse event in any cases. Bleeding requiring endoscopic hemostasis was seen in 2 patients who underwent endoscopic papillectomy and/or endoscopic sphincterotomy. Post-ERCP pancreatitis occurred in 9 cases (4.2%). All but one was mild or moderate in severity and was treated conservatively. There was no need during any procedure to change endoscopes or the stiffness when the TJF-Y0001 was used.

## 5. Discussion

In the present study, we could not demonstrate superiority of a prototype variable stiffness duodenoscope over conventional duodenoscopes. Theoretically, the variable stiffness endoscope should be more useful in certain situations. The variable stiffness duodenoscope should facilitate reaching the major papilla. However, since the distance to the major papilla is relatively short and anatomically uncomplicated as compared to the colon, there does not appear to be a great need for adjustable flexibility during ERCP. Nonetheless, we found that when the papilla could not be reached in a patient with severe duodenal stenosis, the variable stiff duodenoscope was useful as it prevented looping of the endoscope in the stomach. However, care should be taken when a relatively stiff duodenoscope is used to avoid perforation.

A stiffer duodenoscope may facilitate removal of relatively large stones from the bile duct, or placement of large caliber stents across tight strictures. 

Although we excluded cases of Billroth II gastrectomy or Roux-en-Y anastomosis, variable stiffness duodenoscopes may be useful in reaching the papilla or anastomotic site because loop formation can also be prevented or sharp angulation of the small intestine could be overcome. 

The variable stiffness colonoscope has theoretical advantages over standard adult colonoscopies; its usefulness remains controversial [4–6]. One meta-analysis of randomized controlled trials concluded that when variable stiffness colonoscopes were used, higher cecal intubation rates, less abdominal pain, and a decreased need for sedation were seen compared to standard adult colonoscopes, though cecal intubation times were similar [7]. The usefulness of a variable stiffness duodenoscope remains unclear since intubation of the second duodenum during ERCP is usually not difficult and not associated with need for additional sedation. 

In conclusion, the variable stiffness duodenoscope performed similarly to standard duodenoscopes for routine diagnostic and therapeutic ERCP. Further prospective studies in a large number of patients are needed to identify patients who might benefit from this new technology. 

## Figures and Tables

**Table 1:** Basic specifications of each duodenoscope.

Type of duodenoscope	TJF-Y0001	JF-260V	TJF-260V
Field of view, degrees	100°	100°	100°
Depth of field, mm	5 to 60	5 to 60	5 to 60
Distal end outer diameter, mm	13.5	12.6	13.5
Insertion tube diameter, mm	11.6	11.3	11.3
Working length of insertion tube, mm	1240	1240	1240
Length of variable stiffness, mm	26	0	0
Working channel diameter, mm	4.2	3.7	4.2

**Table 2:** Characteristics of patients of each duodenoscope.

Type of duodenoscope	TJF-Y0001	JF-260V	TJF-260V
Number of ERCP sessions	86	60	67
Mean age, years	61.3	65.4	60.5
Gender, men/women	48/38	35/25	33/34
Number of prior Billroth-I gastrectomy	1	1	0
Periampullary diverticulum, %	23%	33%	21%
Number of duodenal stenoses (benign/malignant)	4 (2/2)	2 (0/2)	3 (2/1)

ERCP, endoscopic retrograde cholangiography.

**Table 3:** Procedures performed with each duodenoscope.

Type of duodenoscope	TJF-Y0001	JF-260V	TJF-260V
Sphincterotomy*	52	34	41
Papillary balloon dilation	1	3	4
Stent insertion (exchange)	26	19	21
Nasal-biliary drainge	8	10	6
Nasal-pancreatic duct drainage	0	3	2
Stone extraction	38	33	28
Endoscopic papillectomy	1	0	2
Biopsy (± brushing cytology)	26	22	18
Intraductal ultrasonography	17	22	19
Hemostasis (clipping, HSE injection)	1	1	0

*Including precutting; HSE, hypersaline epinephrine.

## Abbreviations

ERCP: endoscopic retrograde cholangiopancreatography.
